# Low‐dose maternal alcohol consumption: effects in the hearts of offspring in early life and adulthood

**DOI:** 10.14814/phy2.12087

**Published:** 2014-07-31

**Authors:** Vivian B. Nguyen, Megan E. Probyn, Fiona Campbell, Kom V. Yin, Chrishan S. Samuel, Monika A. Zimanyi, John F. Bertram, Mary Jane Black, Karen M. Moritz

**Affiliations:** 1 Department of Anatomy and Developmental Biology, Monash University, Melbourne, Victoria, Australia; 2 School of Biomedical Sciences, University of Queensland, Brisbane, Queensland, Australia; 3 Department of Pharmacology, Monash University, Melbourne, Victoria, Australia

**Keywords:** Cardiomyocyte, ethanol, heart, prenatal, rat

## Abstract

High alcohol consumption during pregnancy leads to deleterious effects on fetal cardiac structure and it also affects cardiomyocyte growth and maturation. This study aimed to determine whether low levels of maternal alcohol consumption are also detrimental to cardiomyocyte and cardiac growth in the early life of offspring and whether cardiac structure and function in adulthood is affected. Pregnant Sprague–Dawley rat dams were fed a control or 6% (volume/volume) liquid‐based ethanol supplemented (isocaloric) diet throughout gestation. At embryonic day 20, the expression of genes involved in cardiac development was analyzed using Real‐time PCR. At postnatal day 30, cardiomyocyte number, size, and nuclearity in the left ventricle (LV) were determined stereologically. In 8‐month‐old offspring, LV fibrosis and cardiac function (by echocardiography) were examined. Maternal ethanol consumption did not alter gene expression of the cardiac growth factors in the fetus or cardiomyocyte number in weanling offspring. However, at 8 months, there were significant increases in LV anterior and posterior wall thickness during diastole in ethanol‐exposed offspring (*P *= 0.037 and *P *= 0.024, respectively), indicative of left ventricular hypertrophy; this was accompanied by a significant increase in fibrosis. Additionally, maximal aortic flow velocity was significantly decreased in ethanol‐exposed offspring (*P *= 0.035). In conclusion, although there were no detectable early‐life differences in cardiac and cardiomyocyte growth in animals exposed to a chronic low dose of ethanol during gestation, there were clearly deleterious outcomes by adulthood. This suggests that even relatively low doses of alcohol consumed during pregnancy can be detrimental to long‐term cardiac health in the offspring.

## Abstract (precis)

Low dose alcohol consumption during gestation in rats illustrated no differences in cardiomyocyte and cardiac growth in the offspring in early life. However, in adulthood, induction of left ventricular hypertrophy was observed suggesting low doses of alcohol during pregnancy can be detrimental to long‐term cardiac health in offspring.

## Introduction

Cardiovascular disease is a leading cause of death and disability worldwide, however, many of the underlying etiological mechanisms remain elusive. Importantly, studies over the past two decades have demonstrated that the antecedents of cardiovascular disease can originate early in life as a result of adaptations made by the developing fetus in response to perturbations *in utero* (Barker et al. 1993; Barker 1995; Hocher 2007). Animal models have demonstrated that insults such as poor maternal diet or uteroplacental insufficiency can alter cardiac development, resulting in a reduction in functional cardiomyocytes (Corstius et al. 2005; Black et al. 2012). It is well recognized that prolonged exposure to high doses of alcohol in utero perturb the development of the heart, commonly leading to congenital heart defects (Loser and Majewski 1977; Webster et al. 1984). In addition, at a cellular level, exposure to moderate or high levels of alcohol in utero can lead to deleterious effects on cardiomyocyte growth and maturation. A recent study in sheep showed that in utero exposure to moderate–high levels of ethanol (EtOH) during late gestation leads to altered growth of cardiomyocytes in newborn lambs; there was a higher percentage of binucleated cardiomyocytes (indicative of altered maturation) and an increase in cardiomyocyte size (Goh et al. 2011). Also, in vitro studies report that EtOH depresses DNA and protein synthesis thus slowing expansion of cardiac myocyte populations in culture (Adickes et al. 1993).

Although there is substantial evidence demonstrating that high levels of maternal alcohol consumption can be teratogenic and deleterious to growth of fetal organs, the effect of in utero exposure to low doses of alcohol on the developing fetus remains unclear. This is important and relevant to many pregnancies; although many women choose to abstain from alcohol consumption during pregnancy, there remains a high proportion of women who continue to consume low to moderate levels of alcohol while pregnant (Colvin et al. 2007; Wallace et al. 2007). For instance, data from the Danish National Birth Cohort from 1996 to 2003, reported out of over 91,000 pregnancies, only 55.4% of women abstained from consuming alcohol during pregnancy (Andersen et al. 2012). Certainly, up until recently, in countries such as Australia the advice given to women was that it was safe to consume low levels of alcohol during pregnancy.

Given that exposure to high and moderate doses of alcohol can lead to marked alterations in growth of cardiomyocytes in the fetal heart, it is imperative to gain an understanding of how exposure to chronic low doses of alcohol during pregnancy affects cardiomyocyte growth in the fetal heart. Hence, in this study, using a rat model of ad libitum 6% EtOH consumption throughout pregnancy (to obtain a plasma EtOH concentration of ~0.03%) (Probyn et al. 2012), the aims were to determine the effects of chronic low dose EtOH exposure on the expression of genes involved in cardiac development at embryonic day 20 (E20), cardiomyocyte growth parameters at weaning (postnatal day 30) (PN30), and cardiac function and structure in adulthood (8 months of age).

## Methods

### Ethics approval

All experimental procedures involving animals were approved by the Animal Welfare Unit of The University of Queensland and followed the Australian Code of Practice for the Care and Use of Animals for Scientific Purposes (7th Edition, National Health and Medical Research Council, 2004).

### Animal groups

Female Sprague–Dawley rats, of at least 8 weeks of age, were habituated to the control liquid diet prior to mating. Female rats were mated overnight and pregnancy confirmed by the presence of a seminal plug. Dams were then allocated to receive either a liquid‐based diet containing 6% v/v EtOH (*n *= 24 dams) or an isocaloric control diet free of EtOH (*n *= 22 dams). These liquid‐based diets were provided fresh daily to dams on an ad libitum basis, until the dams were culled for fetal tissue collection at E20 (*n *= 11 EtOH and 10 control dams) or until they had spontaneously delivered their pups. As reported previously (Probyn et al. 2012), dams in both groups consumed similar amounts of the diets and thus, equal calories, over the duration of pregnancy. Plasma EtOH concentrations of the dams (measured by an EnzyChrome assay kit; BioAssay systems, Hayward, CA) were 0.02 ± 0.01% 15 min after being offered the fresh diet and 0.03 ± 0.01% after 30 min. Plasma EtOH concentrations were undetectable after 60 min. After delivery, the liquid diets were replaced with standard rat chow and water ad libitum. Offspring were nursed by their mothers and weaned on postnatal day 28. At PN30, a subset of offspring (one male and female per litter) was culled and cardiomyocyte growth and maturation were examined. A second subset of offspring (one male and female per litter) was studied at 8 months of age for assessment of in vivo cardiac function and subsequent analyses of left ventricular collagen content. It is important to note that the animals (dams and offspring) used in this study were those used in our previous publication (Probyn et al. 2012) where we described the animal model in detail.

### Embryonic day 20

At E20 (a time point when the fetal heart is rapidly growing and the cardiomyocytes are actively proliferating), a subset of dams and their fetuses were killed as described previously (Probyn et al. 2012). Fetal hearts (one male and one female per litter per treatment group) were weighed and frozen in liquid nitrogen and real‐time PCR used to determine the relative mRNA expression levels of apoptotic genes (*Bax, Bcl‐2* and *p53*), proliferation markers (*c‐Myc, PCNA and Ki67*), cardiomyocyte differentiation genes, (*Gata4* and *Myh10*) and growth‐promoting genes (*Igf‐1)* and its receptor (*Igf1R*), *Igf‐2*,* At1a* receptor (*At1aR*), *At1b* receptor (*At1bR*), *At2* receptor (*At2R*), *FGF‐2* and its receptor (*FGF‐R1*) and *VEGF‐A* and its major receptor, *KDR*. Total RNA was extracted using an RNeasy mini kit (Qiagen, Chadstone, Vic., Australia), then DNase treated and transcribed into cDNA using the Taqman Reverse Transcription Reagents Kit (Applied Biosystems, Carlsbad, CA). *Bax*,* Bcl‐2*,* p53, VEGF‐A, KDR, Ki67, PCNA, FGF‐2, and FGF‐R1* were analyzed using Assays on Demand primer/probe sets (Applied Biosystems); gene expression levels for all other genes were measured using custom‐made primer and probe sequences (Table 1). Quantitation of relative gene expression was measured in a StepOne^™^ Real‐Time PCR system (Applied Biosystems, Mulgrave, Vic., Australia) using the following parameters: initialization – 2 min at 50°C, polymerase activation – 95°C for 10 min, and 40 cycles of denaturation – 15 sec at 95°C and annealing/extension – 1 min at 60°C. The resulting curves were analyzed using the comparative threshold (∆∆*C*_T_) method with ribosomal 18S as the housekeeping gene, as previously described (Singh et al. 2007). The *C*_T_ value for 18S was subtracted from the *C*_T_ value of the gene of interest in each sample to generate a ∆*C*_T_. The mean ∆*C*_T_ of the male control group (used as a calibrator) was then subtracted from each individual sample to give a ∆∆CT value which was inserted into the formula 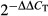. Thus, all samples are expressed relative to the expression levels in the male control group.

**Table 1.** Real‐time PCR primer and Taqman probe sequences.

Gene	Sequence (5′ to 3′)	Genbank accession
c‐Myc		NM_012603
Forward	TGTATGTGGAGCGGCTTCTC	
Reverse	CCTGGTAAGAGGCCAGCTTC	
Probe	CCGCTGCCAAACTGGTCTCCG	
Gata4		NM_144730
Forward	GAGATGCGCCCCATCAAG	
Reverse	GACACAGTACTGAATGTCTGGGACAT	
Probe	CTGTCATCTCACTCTGGGCACAGCAGCTC	
Myh10		NM_031520
Forward	GGCAGCCATCACAGTGACTTC	
Reverse	TGAATTGAGGAGGGAGGGC	
Probe	TGGTCTCTGAGTGTCTGGCTTGATGA	
Igf1		NM_001082477
Forward	GCATTGTGGATGAGTGTTGCT	
Reverse	CAGCGGACACAGTACATCTCC	
Probe	CCGGAGCTGTGATCTGAGGAGGCT	
Igf2		NM_031511
Forward	TACCTCTCAGGCCGTACTTCC	
Reverse	TCCAGGTGTCGAATTTGAAGA	
Probe	CCCCAGATACCCCGTGGGCAA	
Igf1R		NM_052807
Forward	AAGGATGGCGTCTTCACCA	
Reverse	GAGTGGCGATCTCCCAGAG	
Probe	TCATTCCGATGTCTGGTCCTTTGGG	
At1aR		NM_030985
Forward	ATTCCCCCAAAGG CCAAGT	
Reverse	TTATCCGAAGGCCGGTAAGA	
Probe	TCAAGCCTGTCTACGAAAATGAGCACGC	
At1bR		X64052
Forward	TCATTCAGCTGGGCATTATCC	
Reverse	GATGGTGATGGGCATAGCG	
Probe	TGAAGTCTCGCCTCCGCCGC	

Optimal concentrations for primers/probes are 300nM/100nM respectively. c*‐Myc*, myelocytomatosis oncogene; *Gata4*, GATA binding protein 4; *Myh10*¸ myosin, heavy chain; *Igf1*, insulin‐like growth factor 1; *Igf2*, insulin‐like growth factor 2; *Igf1R*, insulin‐like growth factor 1 receptor; *At1aR*, angiotensin II type 1A receptor; *At1bR*, angiotensin II type1B receptor.

### Following weaning – postnatal day 30 (PN30)

In the PN30 offspring, hearts were collected and fixed from nine males and nine females in the ethanol group and eight males and eight females in the control group; cardiomyocyte growth and maturation in the left ventricle and septum were then examined.

#### Heart muscle preparation and sampling

Hearts immersion fixed in 4% paraformaldehyde were weighed after removal of fat and connective tissue. The atria were then removed and the right ventricle (RV) and left ventricle with adjoining septum (LV+S) were weighed separately. The ventricles were cut into 1 mm thick slices and wall volume determined using the Cavalieri principle (Gundersen et al. 1999). Using a smooth fractionator approach, the LV+S slices were sampled for stereological analyses as described previously (Gundersen 2002). These selected slices were embedded in glycolmethacrylate, for the estimation of cardiomyocyte number, with the remaining slices embedded in paraffin for the assessment of cardiomyocyte size and nuclearity.

#### Estimation of cardiomyocyte number

Glycolmethacrylate blocks were serially sectioned at 20 *μ*m with every 10th section collected and stained with Harris’ haematoxylin using a 1000‐watt microwave oven set at 50% power for approximately 2–4 min (Corstius et al. 2005; Lim et al. 2010). Cardiomyocyte number was estimated using an optical disector/fractionator approach (Bruel and Nyengaard 2005; Corstius et al. 2005). Every fourth section was uniformly and systematically sampled. At ×100 magnification, an unbiased counting frame (area of 316.6 *μ*m^2^) was superimposed over the field of view using the C.A.S.T (Computer Aided Stereological Toolbox) program (Olympus, Denmark). Nuclei were counted within a 10 *μ*m depth in the middle of the 20 *μ*m section. The total number of nuclei in the LV+S was estimated by multiplying the number of nuclei counted by the inverse of all the sampling fractions. The total number of cardiomyocytes was then calculated based on the proportion of mono‐, bi‐, tri‐ and, tetra‐nuclear cardiomyocytes (Corstius et al. 2005).

#### Cardiomyocyte size (cross‐sectional area)

**Figure 1.**
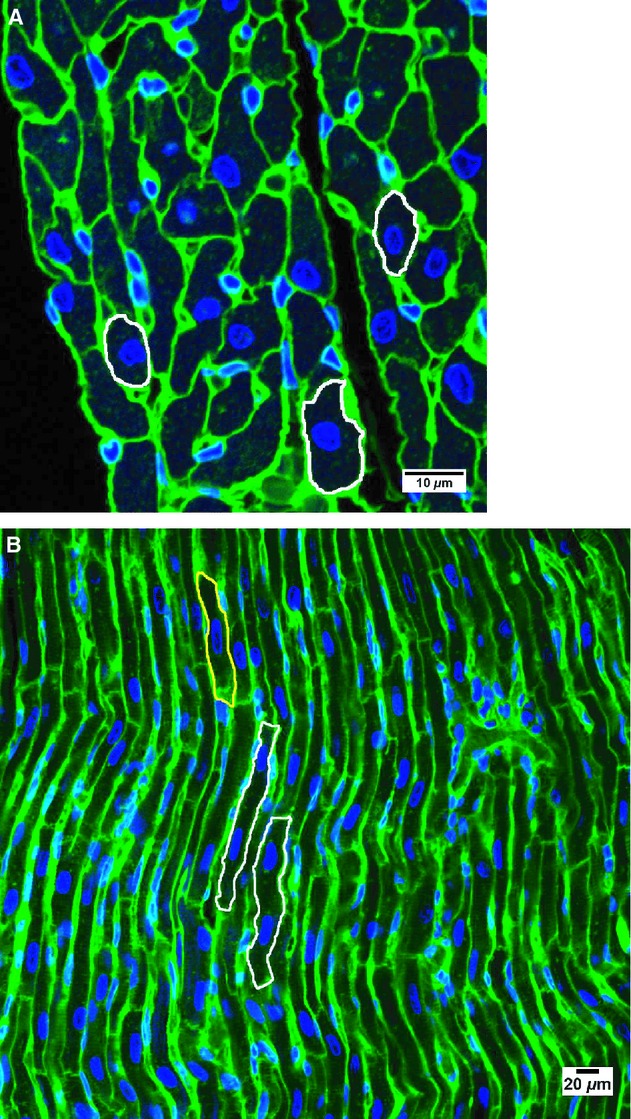
Representative images of cardiomyocytes in a LV+S section stained with wheat germ agglutinin‐Alexa Fluor 488 conjugate (staining cell boundaries green) and DAPI (staining nuclei blue). (A) Only cardiomyocytes in a cross‐sectional plane with a fully intact cell membrane and centrally located nuclei had their boundaries traced (examples outlined in white) and cross‐sectional area determined. Scale bar is 10 *μ*m. (B) Two representative binucleated cardiomyocytes are outlined in white and a mononucleated cardiomyocyte is outlined in yellow. Scale bar is 20 *μ*m.

Paraffin‐embedded samples were sectioned at 5 *μ*m thickness and stained with wheat germ agglutinin‐Alexa Fluor 488 conjugate (Molecular Probes Invitrogen, Mulgrave, Vic., Australia) to stain cell boundaries and 4′6‐diamidino‐2‐phenylindole, dihydrochloride (DAPI) (Molecular Probes Invitrogen) to stain nuclei (Goh et al. 2011). Using NIS‐Elements software (Nikon, Tokyo, Japan), cell boundaries were traced and the cross‐sectional area of cardiomyocytes was measured. Only cardiomyocytes where the nuclei could be seen in the center and the entire cell membrane was intact were traced (see Fig. 1A). The cross‐sectional area of ~150 cardiomyocytes from each animal was measured.

#### Cardiomyocyte nuclearity

The nuclearity of cardiomyocytes (i.e. the number of nuclei in each cardiomyocyte) in the LV+S of each heart was assessed. Paraffin‐embedded LV+S samples were sectioned at 40 *μ*m thickness and stained with wheat germ agglutinin‐Alexa Fluor 488 conjugate and DAPI to stain cell boundaries and nuclei, respectively. Using confocal microscopy, the sections were systematically sampled and images were captured. Utilizing three‐dimensional software (Imaris Version 6.1/6.2; Bitplane, Zurich, Switzerland), the number of nuclei within the cardiomyocytes was then counted in approximately 200 cells per animal. Only cardiomyocytes with a fully intact and visible cell membrane were examined (see Fig. 1B). Cardiomyocytes were recognized by the appearance of prominent long, oval nuclei.

### In adulthood – 8 months of age

#### Echocardiography

Echocardiographic examination (Phillips iE33 with 15 MHz linear array transducer; Phillips Healthcare, Andover, MA) was performed on animals at 8 months of age (10 males and 11 females from the ethanol group and 10 males and 10 females from the control group). Anaesthesia was induced and maintained with inhalant isoflourane (2%) in 100% oxygen administered via a nose cone, attached to an open‐circuit inhalation anesthetic apparatus. Once anesthetized, the rat was placed supine; the thoracic hair was shaved and warmed coupling gel applied to facilitate transducer coupling. Dimensional measurements of the LV were made from a right parasternal short axis view at the level of the papillary muscles from 2‐D guided m‐mode images. Measurements were taken in accordance with the guidelines of the American Society of Echocardiography using the leading‐edge method (Sahn et al. 1978) and included diastolic LV anterior wall dimension (LVAWd), diastolic LV posterior wall dimension (LVPWd) and the internal diameter of the LV in diastole and systole (LVIDd and LVIDs respectively). Pulsed‐wave Doppler spectral profiles, obtained from the left apical 5‐chamber view, with sample volume between LV inflow and outflow were used to assess isovolumic relaxation time (IVRT) and the time between mitral valve (MV) closure and MV opening (MVc‐MVo). Suprasternal 2‐D guided imaging of the ascending aorta facilitated acquisition of pulsed‐waved Doppler spectral profiles of LV outflow from which isovolumic contraction time (IVCT), aortic ejection time (AoET), the aortic velocity time integral (AoVTI), and maximal aortic flow velocity (AoVmax) were measured. From the suprasternal position, aortic diameter was measured at the junction of the ascending and aortic arch during systole for calculation of aortic cross‐sectional area (AoCSA). R–R interval (time between two consecutive QRS complexes on the simultaneously recorded ECG) was measured on the simultaneous ECG during the same cardiac cycles used to obtain the pulsed‐wave Doppler measurements.

Established formulae (Feigenbaum 1989) were used to calculate several indices from measured parameters. From LVIDd and LVIDs, fractional shortening (FS%) was calculated. Cardiac output was calculated from the product of AoCSA, AoVTI, and heart rate. Myocardial performance index (MPI) was determined by dividing total isovolumic times (i.e. MVc‐MVo minus AoET) by AoET.

All measurements were obtained from 3 to 5 consecutive cardiac cycles and averaged.

#### Postmortem analysis

One week after the echocardiography, 8–month‐old offspring were culled and immersion‐fixed hearts were stored in 10% buffered formalin. Fixed hearts were weighed whole after removal of fat and connective tissue. The atria and right ventricle were then removed and the left ventricle and septum (LV+S) weighed.

#### Hydroxyproline analysis

Equivalent portions of LV tissue from 8‐month‐old offspring (seven males and nine females from the ethanol group and eight males and six females from the control group) were assessed for their hydroxyproline content to estimate collagen concentration (a measure of fibrosis), as previously described (Samuel et al. 1996). Duplicate 10 *μ*L aliquots from each sample were analyzed for hydroxyproline content using a scaled‐down version of the method described by Bergman and Loxley (Bergman and Loxley 1963). Hydroxyproline values were then converted to collagen content by multiplying by a factor of 6.94 (as hydroxyproline represents ~14.4% of the amino acid composition of collagen in most mammalian tissues (Gallop and Paz 1975), and then divided by the dry weight of each respective tissue portion to yield % collagen concentration.

### Statistical analysis

Statistical analyses were performed and graphed using GraphPad Prism Version 5.0 (GraphPad Software, San Diego, CA). All data were analyzed using a two‐way analysis of variance (ANOVA), with sex and alcohol prenatal exposure as factors. Data are represented as means ± standard error of the mean (SEM), and statistical significance was accepted at the level of *P* < 0.05.

## Results

### Embryonic day 20

#### Body and heart weights

EtOH‐exposed male and female fetuses were significantly smaller than controls on a whole litter basis (Probyn et al. 2012), however, in the subset of fetuses randomly selected for this study, there were no differences in body weight compared to same sex controls (Table 2). Similarly, there were no differences in heart weights (absolute or relative to body weight) between male or female control or EtOH‐exposed fetuses.

**Table 2.** Fetal body and heart weight on embryonic day 20 and relative expression of genes involved in cardiac development.

	Male		Female		*P* values
	Control (*n *= 11)	EtOH (*n *= 11)	Control (*n *= 10)	EtOH (*n *= 12)	
Body wt (g)	2.49 ± 0.07	2.57 ± 0.06	2.62 ± 0.11	2.43 ± 0.07	NS
Heart wt (mg)	15.3 ± 0.6	16.3 ± 0.9	14.1 ± 1.6	15.7 ± 1.1	NS
Heart wt (mg/g BW)	6.2 ± 0.3	6.3 ± 0.3	5.4 ± 0.6	6.4 ± 0.4	NS
Gene expression					
* Bax*	1.12 ± 0.24	0.81 ± 0.08	1.26 ± 0.21	0.88 ± 0.10	NS
* Bcl‐2*	1.02 ± 0.13	1.17 ± 0.20	2.38 ± 0.83	1.30 ± 0.36	NS
* c‐Myc*	1.14 ± 0.14	1.35 ± 0.18	1.80 ± 0.41	1.14 ± 0.20	NS
* PCNA*	1.15 ± 0.19	1.03 ± 0.15	1.60 ± 0.27	0.79 ± 0.13	NS
* Ki67*	1.12 ± 0.16	1.16 ± 0.17	1.71 ± 0.37	1.01 ± 0.19	NS
* p53*	1.18 ± 0.23	1.08 ± 0.13	2.63 ± 1.04	1.24 ± 0.30	NS
* Gata4*	1.15 ± 0.17	2.42 ± 0.58	2.54 ± 0.67	1.98 ± 0.54	NS
* Myh10*	1.09 ± 0.18	0.93 ± 0.17	1.16 ± 0.21	1.13 ± 0.19	NS
* Igf1*	1.13 ± 0.19	0.65 ± 0.14	2.02 ± 0.62	0.60 ± 0.13	NS
* Igf2*	1.09 ± 0.16	1.07 ± 0.16	1.48 ± 0.35	1.16 ± 0.26	NS
* Igf1R*	1.03 ± 0.13	1.12 ± 0.17	1.34 ± 0.29	1.04 ± 0.19	NS
* At1aR*	1.51 ± 0.24	1.78 ± 0.21	1.58 ± 0.40	1.46 ± 0.22	NS
* At1bR*	1.10 ± 0.21	0.87 ± 0.16	2.11 ± 0.91	1.20 ± 0.32	NS
* FGF‐2*	1.18 ± 0.22	1.40 ± 0.17	1.43 ± 0.31	1.14 ± 0.19	NS
* FGF‐R1*	1.09 ± 0.15	1.22 ± 0.11	1.36 ± 0.21	1.21 ± 0.16	NS
* VEGF‐A*	1.29 ± 0.29	1.30 ± 0.18	1.53 ± 0.43	1.36 ± 0.27	NS
* KDR*	1.04 ± 0.09	1.13 ± 0.10	1.36 ± 0.27	0.93 ± 0.07	NS

Gene expression (relative to 18S and calibrated to the male control group) is expressed in arbitrary units. Numbers in parentheses are group sizes. Data are expressed as mean ± SEM. wt, weight; BW, body weight; NS, not significant. Data analyzed via two‐way ANOVA with sex and treatment as factors.

#### Gene expression of markers associated with cardiac development

At E20, the relative expression levels of genes associated with cardiomyocyte growth, cellular proliferation and apoptosis were not significantly different between EtOH‐exposed and control male and female fetuses.

### Postnatal day 30

#### Body weights and heart growth

At PN30, there were no significant differences between EtOH‐exposed and control offspring in body weight and in absolute and relative heart weights, RV and LV+S weights and RV and LV+S wall volumes (Table 3). Overall, female offspring had lighter absolute and relative LV+S weights and smaller LV wall volumes than male offspring.

**Table 3.** Body weight, absolute and relative heart and ventricular weight, and ventricular volume of offspring culled at postnatal day 30.

	Male		Female		*P* values
	Control (*n *= 8)	EtOH (*n *= 9)	Control (*n *= 8)	EtOH (*n *= 9)	
Body wt (g)	86.4 ± 3.0	81.1 ± 3.8	80.2 ± 2.8	75.9 ± 2.8	NS
Absolute					
Heart wt (g) (fixed)	0.395 ± 0.025	0.378 ± 0.216	0.362 ± 0.022	0.336 ± 0.017	NS
LV+S wt (g)	0.242 ± 0.011	0.231 ± 0.011	0.211 ± 0.008	0.202 ± 0.009	*P*_S_ = 0.006
RV wt (g)	0.058 ± 0.001	0.054 ± 0.003	0.052 ± 0.003	0.054 ± 0.004	NS
LV wall volume (mm^3^)	262.125 ± 10.528	256.500 ± 10.706	236.250 ± 9.249	224.250 ± 10.392	*P*_S_ = 0.008
RV wall volume (mm^3^)	93.656 ± 3.505	93.500 ± 4.006	88.875 ± 4.619	90.750 ± 5.114	NS
Relative to body wt					
Heart wt (mg/g)	4.563 ± 0.219	4.665 ± 0.216	4.544 ± 0.267	4.417 ± 0.175	NS
LV+S wt (mg/g)	2.797 ± 0.067	2.858 ± 0.088	2.644 ± 0.091	2.669 ± 0.084	*P*_S_ = 0.049
RV wt (mg/g)	0.674 ± 0.024	0.669 ± 0.020	0.648 ± 0.041	0.708 ± 0.038	NS
LV wall volume (mm^3^/g)	3.036 ± 0.077	3.190 ± 0.118	2.968 ± 0.133	2.959 ± 0.101	NS
RV wall volume (mm^3^/g)	1.091 ± 0.049	1.165 ± 0.051	1.117 ± 0.065	1.193 ± 0.045	NS

Heart weight was recorded when fixed with formalin. wt, weight; LV+S, left ventricle plus septum; RV, right ventricle; NS, not significant. Data were analyzed via two‐way ANOVA with sex (*P*_S_) and treatment (*P*_T_) as factors.

#### Cardiomyocyte growth

**Figure 2.**
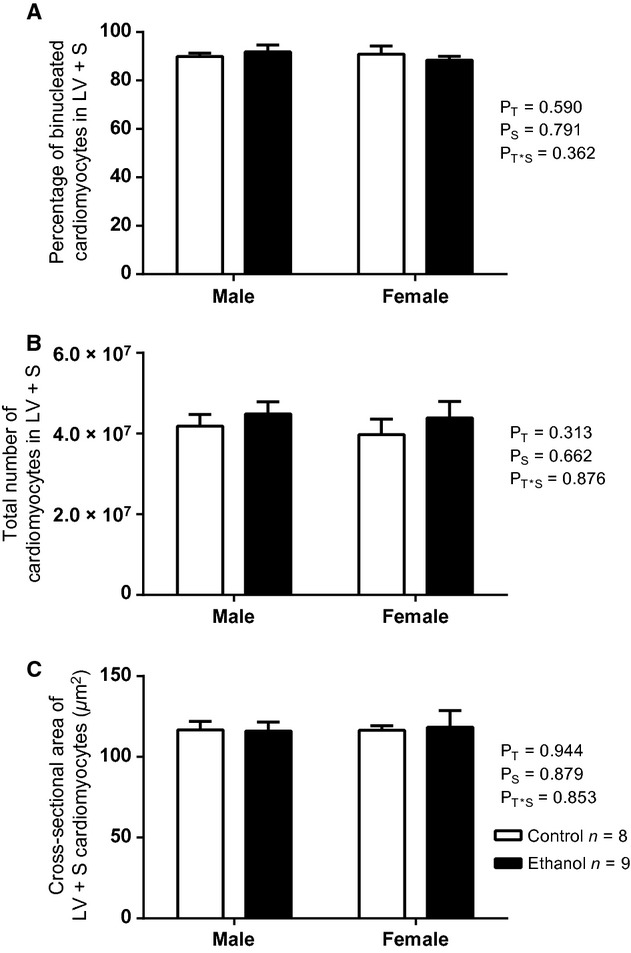
The proportion of binucleated cardiomyocytes (A), total number of cardiomyocytes (B) and cardiomyocyte cross‐sectional area (C) in the LV+S of male and female EtOH‐exposed and control hearts of rat offspring at postnatal day 30. Data analyzed via two‐way ANOVA with sex (*P*_S_) and treatment (*P*_T_) as factors.

For all groups, the majority of cardiomyocytes within the LV+S were binucleated (≥88%) with ≤10% of cardiomyocytes being mononucleated. Occasionally, trinucleated and tetranucleated cardiomyocytes were observed (≤2%). There were no statistical differences in the proportions of mono‐, bi‐ (Fig. 2A), tri‐, or tetra‐nucleated cardiomyocytes within the hearts of EtOH‐exposed and control male or female offspring.

At PN30, the total number of cardiomyocytes within the LV+S was not significantly different between EtOH‐exposed and control male or female offspring (Fig. 2B). This finding was not changed when LV+S cardiomyocyte number was corrected for body weight, heart weight, LV+S weight, or LV+S volume. The mean cross‐sectional area of the LV+S cardiomyocytes was not significantly different between the four groups (Fig. 2C).

### Adulthood – 8 months

#### Body and heart weights

At 8 months of age, there were no differences in body weights between control and EtOH‐exposed offspring, although males were heavier than females (*P* < 0.001). There was an increase in absolute and relative heart weight to body weight in animals that had been exposed to EtOH (Table 4). This was most prominent in females where heart weight relative to body weight was increased by ~20% compared to a ~8% increase in males. There was no significant difference in absolute LV+S weights between groups, however, relative to body weight, there was a significant increase in offspring exposed to EtOH.

**Table 4.** Body weight, absolute and relative heart and ventricular weight, and echocardiography analyses (excluding those shown in Figs. 4, 5) of offspring culled at 8 months of age.

	Male		Female		*P* values
	Control (*n *= 10)	EtOH (*n *= 10)	Control (*n *= 10)	EtOH (*n *= 11)	
Body wt (g)	586 ± 22	574 ± 19	337 ± 13	322 ± 9	*P*_S_ < 0.001
Absolute					
Heart wt (g) (fixed)	2.06 ± 0.10	2.19 ± 0.09	1.31 ± 0.06	1.48 ± 0.04	*P*_T_ = 0.024 *P*_S_ < 0.001
LV+S wt (g)	1.32 ± 0.06	1.36 ± 0.04	0.86 ± 0.04	0.93 ± 0.02	*P*_S_ < 0.001
Relative to body wt					
Heart wt (g/g)	3.54 ± 0.12	3.81 ± 0.12	3.85 ± 0.16	4.63 ± 0.19	*P*_T_ < 0.001 *P*_S_ < 0.001
LV+S wt (mg/g)	2.26 ± 0.08	2.38 ± 0.06	2.53 ± 0.07	2.89 ± 0.07	*P*_T_ = 0.006 *P*_S_ < 0.001
Echocardiography					
LVIDd (mm)	8.1 ± 0.2	8.0 ± 0.3	6.3 ± 0.2	6.6 ± 2.2	*P*_S_ < 0.001
LVIDs (mm)	4.9 ± 0.2	4.5 ± 0.3	3.6 ± 0.2	3.9 ± 0.2	*P*_S_ < 0.001
IVCT (msec)	22.4 ± 0.6	23.0 ± 0.9	22.0 ± 1.0	21.5 ± 0.7	NS
IVRT (msec)	24.8 ± 1.2	25.6 ± 1.8	24.4 ± 0.9	24.2 ± 0.8	NS
RR interval (msec)	171.3 ± 6.8	167.6 ± 4.6	171.9 ± 4.6	180.0 ± 5.5	NS
MVc‐MVo (msec)	99.3 ± 1.8	99.8 ± 2.1	97.9 ± 2.7	98.4 ± 1.8	NS
Ao CSA	10.3 ± 0.4	11.3 ± 0.8	8.0 ± 0.4	7.6 ± 0.4	*P*_S_ < 0.001
Ao diameter (mm)	3.6 ± 0.1	3.8 ± 0.1	3.2 ± 0.1	3.1 ± 0.1	*P*_S_ < 0.001
Ao VTI (cm)	5.6 ± 0.3	4.4 ± 0.2	4.4 ± 0.2	4.5 ± 0.3	*P*_S_ = 0.027
Ao Vmax (m/s)	1.06 ± 0.04	0.89 ± 0.05	0.93 ± 0.03	0.92 ± 0.04	*P*_T_ = 0.035
Ao ET (msec)	76.2 ± 2.1	74.3 ± 1.9	70.0 ± 2.4	73.4 ± 2.3	NS

Heart weight was recorded when fixed with formalin. wt, weight; LVID, left ventricle internal diameter; d, diastole; s, systole; IVCT, isovolumic contraction time; IVRT, isovolumic relaxation time; RR interval, time between two consecutive QRS complexes on the simultaneously recorded ECG; MVc‐MVo, time from mitral valve closure to opening; Ao CSA, aortic cross‐sectional area; Ao VTI, aortic velocity time integral; Ao Vmax, maximal aortic flow velocity; Ao ET, aortic ejection time; msec, milliseconds; NS, not significant. Data were analyzed via 2 way ANOVA with sex (*P*_S_) and treatment (*P*_T_) as factors.

#### Echocardiographical analyses

**Figure 3.**
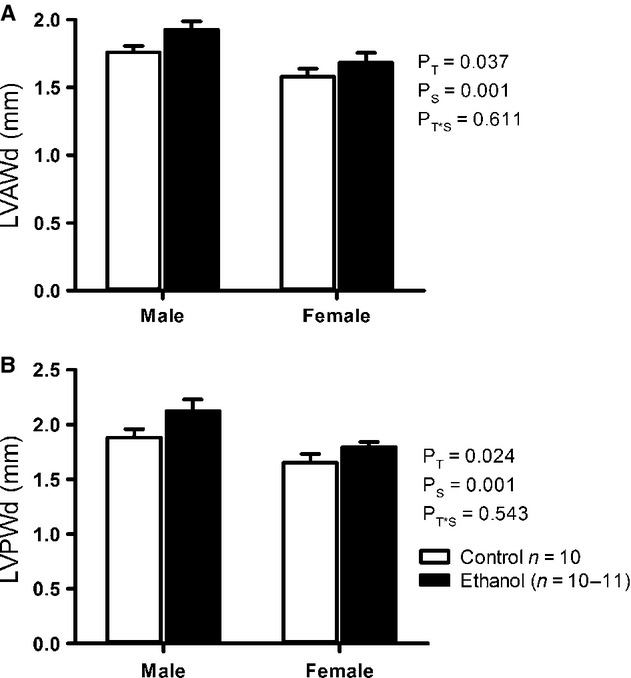
Echocardiographical measurements of left ventricular anterior wall thickness during diastole (LVAWd) (A) and left ventricular posterior wall thickness during diastole (LVPWd) (B) in male and female EtOH‐exposed and control rat offspring at 8 months of age. Data analyzed via two‐way ANOVA with sex (*P*_S_) and treatment (*P*_T_) as factors.

LVAWd was significantly thicker (*P* = 0.037) in male (6%) and female (7%) EtOH‐exposed offspring compared to controls (Fig. 3A). Likewise, LVPWd was also significantly thicker (*P* = 0.024) in male and female EtOH‐exposed offspring (10% and 6% respectively) compared to controls (Fig. 3B). There was no difference between treatment groups in LVIDs and LVIDd; however, there was a sex effect with these values being higher in males compared to females (Table 4).

**Figure 4.**
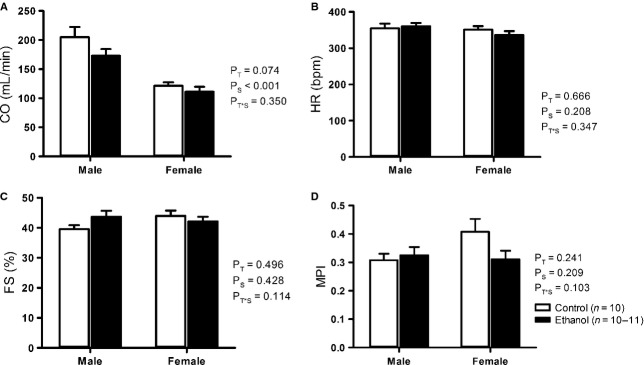
Echocardiographical measurements of cardiac output (CO) (A), heart rate (HR) (B), fractional shortening (FS%) (C) and myocardial performance index (MPI) (D) in male and female EtOH‐exposed and control rat offspring at 8 months of age. Data analyzed via two‐way ANOVA with sex (*P*_S_) and treatment (*P*_T_) as factors.

There was no significant difference in heart rate (Fig. 4B) or cardiac output (Fig. 4A) between groups. The effect of sex was highly significant, with reduced CO in female offspring compared to males (*P* < 0.001). There was no difference in fractional shortening between treatment groups (Fig. 4C). AoVmax was significantly reduced (*P* = 0.035) in male and female EtOH‐exposed offspring compared to controls (Table 4). Prenatal EtOH exposure did not affect any other parameters measured during echocardiography (Table 4).

#### Total left ventricular collagen content

**Figure 5.**
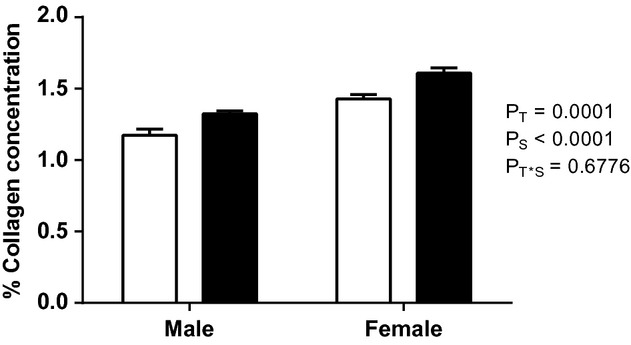
Total collagen content in the LV of male and female EtOH‐exposed and control rat offspring at 8 months of age. Data analyzed via two‐way ANOVA with sex (*P*_S_) and treatment (*P*_T_) as factors.

At 8 months of age, the percentage of collagen in LV tissue was significantly higher (*P* = 0.0001) in the EtOH‐exposed offspring (males: 1.32%; females: 1.61%) compared to controls (males: 1.17%; females: 1.43%). The effect of sex was also highly significant, with a lower percentage of collagen in the LVs in male offspring compared to females (*P* < 0.0001) (Fig. 5).

## Discussion

While high levels of EtOH exposure during development are well documented to have deleterious effects on cardiac development, this is one of the first studies to examine the effects of a more modest EtOH exposure on heart development and on cardiac function later in life. Maternal consumption of a chronic low level of EtOH throughout pregnancy did not lead to any overt differences in the expression of key genes associated with cardiomyocyte growth in the fetal heart. Cardiomyocyte size, number, and nuclearity at weaning were not affected by EtOH exposure, however, by adulthood, adverse effects on cardiac structure manifest, with the development of cardiac and left ventricular hypertrophy and left ventricular fibrosis. This was accompanied by a reduction in maximal aortic flow velocity.

In this study, we found no effect of maternal alcohol consumption on fetal body and heart weights at E20. This is contrary to our previous findings at E20 in this model, where litter averages were reported (Probyn et al. 2012). The discrepancy in findings is likely attributed to the smaller sample size in the present study, where only one male and one female from each litter (chosen at random) was used. It should be noted that the growth restriction previously reported (Probyn et al. 2012), in the larger sample size, was modest (5–7%) and thus it is not surprising that this does not reach statistical significance when the sample size is smaller.

### Exposure to low levels of maternal alcohol consumption had no effect on key genes associated with cardiac development at E20

Given that studies of moderate‐high levels of maternal alcohol consumption led to differences in the expression of genes associated with cardiac development (Gatford et al. 2007; Goh et al. 2011), it was considered likely that in utero exposure to a chronic low dose of alcohol would also impact on cardiac gene expression and cardiac development. The specific genes examined are known to be critically involved in the development of the embryonic heart. We hypothesized that the levels of genes that promote cardiomyocyte proliferation, differentiation, and growth would be reduced, particularly insulin‐like growth factor as this has been previously shown to be decreased after prenatal acute alcohol exposure (Gatford et al. 2007). Alternatively, expression of the pro‐apoptotic genes were expected to increase (Goh et al. 2011). Contrary to our hypothesis, gestational EtOH exposure did not elicit any detectable changes in expression levels of the cardiac growth‐promoting genes or apoptotic genes that we looked at.

These findings are in contrast to previous studies in fetal sheep, where exposure to moderate levels of EtOH (plasma EtOH concentrations reaching a maximum of 0.12%) late in gestation led to a marked upregulation of mRNA expression of the cardiomyocyte growth factor *Igf1* and apoptotic genes, *caspase 3* and *Bax*, in the left ventricle (Goh et al. 2011). The discrepancy in findings between studies may relate to differences in species, the dose of alcohol or alternatively the timing of the insult. Indeed, an acute high dose exposure to alcohol during late gestation is likely to act as an insult to the developing heart (as indicated by upregulation of pro‐apoptotic genes) resulting in a subsequent reactive rise in genes associated with cardiomyocyte growth (Goh et al. 2011). In contrast, the developing heart exposed to a relatively low dose of alcohol from conception (as in the present study) may adapt to a constant exposure to alcohol throughout pregnancy, such that cardiomyocyte growth is not affected. Alternatively, the low dosage used in our study may not have reached a threshold sufficient to elicit changes.

Given the disparity in our findings compared to other studies, in future studies it would be beneficial to conduct a study where the dams consume varying doses of ethanol to examine the dose effects. However, it should also be noted that studies using high doses of ethanol can be confounded by significant changes in neurological and behavioral function (Tewari et al. 1992), which may in turn affect cardiovascular function.

### Growth of the heart and cardiomyocytes appears normal at PN30

As there was no evidence to suggest that chronic low levels of maternal alcohol consumption throughout pregnancy directly affects genes regulating cardiomyocyte proliferation, apoptosis, maturation or differentiation, it is perhaps not surprising that there were no effects on heart size or cardiomyocyte number, size and nuclearity at the time of weaning (a timepoint when the cardiomyocytes have recently stopped proliferating and undergone maturation). A number of studies have shown that the complement of cardiomyocytes at birth is directly dependent on heart size. For instance, it has been demonstrated that the number of cardiomyocytes in the LV+S is significantly and directly related to the heart tissue weights as well as birth weights in 9‐week‐old lambs (Stacy et al. 2009). Similarly, in the rat model, it has been shown that cardiomyocyte number exhibits a significant linear correlation to heart volume at weaning (Lim et al. 2010). Hence, based on heart size, it is also expected that total cardiomyocyte number would not be different between the alcohol exposed and control groups given that heart size was similar at PN30.

When considering our findings, it is important to keep in mind that growth and maturation of cardiomyocytes in the rat heart differs from the human with proliferation ongoing after birth. Hence, in relation to cardiac ontogeny, the timing of EtOH exposure in our rat model is not equivalent to the entire human pregnancy.

### Cardiac structural changes and dysfunction manifests in later life

Given that there were no overt differences in cardiac size or cardiomyocyte growth at PN30, an interesting finding of this study was the induction of cardiac hypertrophy at 8 months of age. This is attributed to hypertrophy of the left ventricle with the anterior and posterior wall of the LV during diastole in both males and females being significantly thicker in EtOH‐exposed offspring compared to controls. This is of major clinical importance, as left ventricular hypertrophy has been shown to be the strongest predictor of adverse cardiovascular events irrespective of age, sex, and blood pressure (Schillaci et al. 2000; Paoletti et al. 2004). Of concern, the left ventricular hypertrophy appears to be due to increased myocardial fibrosis, with a 12.7% increase in total collagen content in the hearts of the EtOH‐exposed offspring. This is suggestive of pathological left ventricular hypertrophy with increased deposition of interstitial collagen linked to stiffening of the ventricular walls (Weber et al. 1990) and impaired conductivity (Spach and Boineau 1997). The mechanisms leading to the induction of the left ventricular hypertrophy in our adult EtOH‐exposed offspring are unknown, but may relate to changes in haemodynamic load as a result of altered peripheral resistance. In this regard, alcohol consumption by rats throughout pregnancy has been previously reported to lead to a significant increase in the systolic blood pressure of offspring at 25 weeks of age and this appeared to be mediated by alterations in endothelial function (Turcotte et al. 2002). In addition, differences in cerebrovascular reactivity in adult sheep offspring following in utero alcohol exposure have been reported (Ngai et al. 2008). Therefore, as follow up to our current findings, in future studies it would be important to measure blood pressure and assess vascular reactivity in adult EtOH‐exposed offspring.

In addition to the induction of left ventricular hypertrophy in adulthood, we observed a decrease in maximal aortic flow velocity in the EtOH‐exposed offspring compared to controls. This is of concern, as it suggests that left ventricular function may be impaired; this is supported by the increased collagen content in the myocardium which is likely to adversely affect cardiac contractility and overall cardiac function. Alternatively, given that the fractional shortening of the cardiac muscle was not impaired in the EtOH‐exposed offspring compared to controls, the decrease in maximal aortic flow velocity in the EtOH‐exposed offspring may be attributed to vascular dysfunction. Further studies are required to elucidate this.

### Perspectives and significance

The findings from this study demonstrate that prenatal exposure to even low doses of alcohol can lead to left ventricular hypertrophy in adulthood. Given that left ventricular hypertrophy is a major risk factor for cardiovascular disease; these clinically important findings support the advice currently given to women to abstain from consuming alcohol during pregnancy. In future studies, analyses of blood pressure and vascular reactivity in offspring exposed to a chronic low dose of alcohol during gestation would provide valuable insight into the mechanisms leading to the induction of left ventricular hypertrophy which occurs post weaning.

## Acknowledgments

We would like to acknowledge the histological assistance and advice of Mr. Jonathan Bensley, Dr. Kyungjoon Lim, Ms. Sue Connell, and Ms. Julie Hickey.

## Conflict of Interest

On behalf of all authors, the corresponding author states that there is no conflict of interest.
